# Factors associated with suicide attempts among Australian transgender adults

**DOI:** 10.1186/s12888-021-03084-7

**Published:** 2021-02-08

**Authors:** Sav Zwickl, Alex Fang Qi Wong, Eden Dowers, Shalem Yiner-Lee Leemaqz, Ingrid Bretherton, Teddy Cook, Jeffrey D. Zajac, Paul S. F. Yip, Ada S. Cheung

**Affiliations:** 1 Trans Health Research Group, Department of Medicine (Austin Health), The University of Melbourne, Melbourne, Victoria 3084 Australia; 2 College of Medicine and Public Health, Flinders University, Adelaide, South Australia 5042 Australia; 3 Department of Endocrinology, Austin Health, 145 Studley Road, Heidelberg, Victoria 3084 Australia; 4 ACON Health, Surry Hills, New South Wales Australia; 5 Centre for Suicide Research and Prevention, The University of Hong Kong, Hong Kong, China

**Keywords:** Transgender, Mental health, Suicide, Depression

## Abstract

**Background:**

Transgender, including gender diverse and non-binary people, henceforth referred to collectively as trans people, are a highly marginalised population with alarming rates of suicidal ideation, attempted suicide and self-harm. We aimed to understand the risk and protective factors of a lifetime history of attempted suicide in a community sample of Australian trans adults to guide better mental health support and suicide prevention strategies.

**Methods:**

Using a non-probability snowball sampling approach, a total of 928 trans adults completed a cross-sectional online survey between September 2017 and January 2018. The survey assessed demographic data, mental health morbidity, a lifetime history of intentional self-harm and attempted suicide, experiences of discrimination, experiences of assault, access to gender affirming healthcare and access to trans peer support groups. Logistic regression was used to examine the risk or protective effect of participant characteristics on the odds of suicide.

**Results:**

Of 928 participants, 85% self-reported a lifetime diagnosis of depression, 63% reported previous self-harm, and 43% had attempted suicide. Higher odds of reporting a lifetime history of suicide attempts were found in people who were; unemployed (adjusted odds ratio (aOR) 1.55 (1.05, 2.29), *p* = 0.03), had a diagnosis of depression (aOR 3.70 (2.51, 5.45), *p* < 0.001), desired gender affirming surgery in the future (aOR 1.73 (1.14, 2.61), *p* = 0.01), had experienced physical assault (aOR 2.01 (1.37, 2.95), *p* < 0.001) or experienced institutional discrimination related to their trans status (aOR 1.59 (1.14, 2.23), *p* = 0.007).

**Conclusion:**

Suicidality is associated with barriers to gender affirming care, gender based victimisation and institutionalised cissexism. Interventions to increase social inclusion, reduce transphobia and enable timely access to gender affirming care, particularly surgical interventions, are potential areas of intervention.

## Background

Transgender, including gender diverse and non-binary (trans) people are a highly marginalised group in our community with alarmingly high rates of suicidality (ideation and non-fatal behaviours) and mental health morbidities [1–3]. High quality empirical evidence and data (such as from a census) describing the size of the trans population are limited, but a systematic review of studies published internationally from 2009 to 2019 found estimates ranged from 0.5 to 4.5% of the adult population [4]. Within an Australian-context, despite universal public health care and anti-discrimination laws at the State and Federal level, trans adults experience high levels of discrimination and are four times more likely than the general population to be diagnosed with depression, with over 40% self-reporting previous suicide attempts [5–7]. Various human rights challenges remain; in many Australian States and Territories, it is not possible to obtain legal gender recognition without first having gender affirmation surgery. Moreover, access to gender affirmation surgery is not covered by the national Medicare public health scheme and is cost prohibitive for many people.

Suicide attempts and suicide deaths occur due to a complex interaction between biological, psychological and psychosocial risk factors. This may include genetic predisposition to depression and anxiety [8, 9], minority stress and stressful life events, unemployment and financial stress [10–12], quality of support networks [13–17], discrimination, violence [18–20] and barriers to accessing healthcare and support services [21].

Trans-specific factors for suicidality is an under-researched area, but several risk and protective factors have been identified. Research has increasingly focused on how cissexism, or the belief that cisgender people are ‘normal’, ‘natural’ and ‘superior’ delimits opportunities for trans health and wellbeing [22]. Gender-based victimisation, including verbal abuse, peer rejection, threats of violence and physical assault has been well documented among trans adults [3, 23, 24]. Similarly, there is growing evidence of institutionalized cissexism, manifesting as heightened rates of trans unemployment, reduced access to housing, education and healthcare (including gender affirming healthcare), which contributes to diminished mental health and wellbeing by way of elevated feelings of shame, hopelessness and isolation [24–29]. Systemic barriers are associated with increased risk of housing instability, financial stress and violence [30].

Rather than focusing on the deleterious effects of cissexism, research has begun to illuminate factors that protect against suicidality and mental health comorbidities. For example, in trans people who wish to access hormones, being able to do so reduces mental distress, and improves quality of life [31, 32]. Similarly, trans adults who desire and are able to access gender affirming surgery report stronger mental health as compared to trans adults who cannot access surgeries [33]. Social support from family, friends and connection with the trans community and experiencing lower levels of structural discrimination are further protective factor against suicidality and suicide attempts [13–17].

Gender plays a role. In Australia, young cisgender men and those presumed to be men who live in non-metropolitan areas have the highest suicide rates and are less likely to seek assistance for depression or other mental health problems [34]. Data from many countries worldwide show that people presumed male have higher rates of suicide compared to people presumed female [35]. The precise reasons for the gender discrepancy are unclear, however possible explanations for higher rates of suicide in people presumed male include more violent, immediately lethal means of suicide, higher levels of suicidal intent and greater reticence to seek assistance from doctors for mental health support [36, 37].

In the general population, it is known that unemployment, physical assault and perceived discrimination increases risk for suicide ideation and suicide attempts [12, 38, 39]. We hypothesised that people who reported known risk factors for suicidal behaviour; residing in rural areas, unemployment, experienced difficulty accessing gender-affirming interventions, known history of depression or anxiety, had perceived discrimination and experiences of assault, would have a higher odds of reporting a history of suicide attempts. Given the lack of data describing risk or protective factors among Australian trans adults, this exploratory analysis aimed to assess factors associated with a lifetime history of attempted suicide in order to guide suicide prevention strategies and interventions.

## Methods

This anonymous online survey of trans adults utilised a non-probability snowball sampling technique. Inclusion criteria for participants were assessed by a positive response to three screening questions: a) Australian residency; b) aged 18 years or older and c) self-identify as trans or gender diverse (defined as a ‘yes’ response to the question ‘Do you currently identify or have you previously identified as transgender or gender diverse?’). The inclusion of those who had previously identified as trans was intended to include those who identified as their affirmed gender (male, female or non-binary) rather than with the term transgender. Individuals were eligible to complete the survey on one occasion only and duplicate responses from the same Internet Protocol address were excluded. All included individuals had discordance between their assigned sex at birth and their gender identity. Survey questions were all optional. SurveyMonkey (SurveyMonkey Inc. San Mateo, California, USA) was used to collect responses to the survey between 1st September 2017 and 31st January 2018. Given that this was an anonymous survey, written informed consent was not possible and was waived by the institutional ethics committee; however, the survey preamble outlined that completion of the survey implied consent. The study was approved by the Austin Health Human Research and Ethics Committee (HREC/17/Austin/372).

Participants were asked a range of questions, with data pertaining to the health care needs and priorities of participants which are published elsewhere [7, 40]. The full version of the survey is available in the supplementary appendix at 10.1089/lgbt.2020.0178 [7]. Participants were asked ‘Have you ever intentionally self-harmed?’ (response options of ‘yes’, ‘no’ or ‘prefer not to say’) and ‘Have you ever attempted suicide?’ with response options of ‘yes’, ‘no’ or ‘prefer not to say’. We specifically assessed if the following 9 factors were risk or protective factors for a positive (‘yes’) response for a lifetime history of attempted suicide.

1. Location of residence (metropolitan or rural), which was determined by coding postcodes as per the Australia Standard Geographical Classification Remoteness Area (RA). Rural location of residence was classified as anyone living outside of a major city area corresponding to Remoteness Areas 2 to 5.
2. Presumed gender at birth (male, female).
3. Employment status (unemployed, compared to employed on full-time basis, part-time basis, home duties full time, student, retired, other)
4. Access to gender affirming hormones. Participants were asked if they experienced any difficulty accessing gender affirming hormones with positive responses to the following multiple choice options: unable to find a doctor to prescribe; unable to afford costs of prescriptions; unable to afford cost of doctors’ appointments; or pathway to accessing hormones too difficult, compared to no difficulty accessing gender affirming hormones.
5. Access to gender affirming surgery. Participants indicated whether they wanted gender affirming surgery someday, had already had surgery or did not want surgery. We selected the 2 most performed surgical procedures in the trans population, which were feminising genital reconstruction for people presumed male at birth, and bilateral mastectomy or chest reconstruction surgery for people presumed female at birth and assessed those that desired gender affirming surgery someday compared with other groups that did not.
6. Self-reported diagnosis of depression. Participants were asked if they had ever been medically diagnosed with depression (yes/no).
7. Access to trans support groups. Participants were asked if they were a member of any trans support groups, including on social media (yes/no or unsure).
8. Perceived discrimination from employment, housing, healthcare and/or government services. Participants were asked ‘Because of your trans status have you ever experienced any of the following (select all that apply)?’ with multiple choice options of ‘Discrimination from employment (i.e. lost a job or overlooked for a job)’, ‘Discrimination from housing (i.e. denied a rental application)’, ‘Discrimination from accessing healthcare’, ‘Discrimination from government services (i.e. Centrelink)’, ‘Physical assault’, ‘Verbal abuse’, ‘Domestic violence’, and ‘None’. For the purposes of analyses positive responses to any of the four discrimination options (discrimination from employment, housing, accessing healthcare and government services) were combined to create one factor called ‘institutional discrimination’.
9. Physical assault. Participants indicated whether they had ever experienced physical assault because of their trans status (yes/no).

Statistical analysis was performed using R version 3.6.3 (R Foundation for Statistical Computing). Participant characteristics are reported as frequency (percentage). Logistic regression was used to estimate the effects of the 9 factors listed above on the risk of attempted suicide. The 9 factors considered in the regression were selected prior to performing the analysis on the basis of previous known risk factors for suicidal behaviour. Results are reported as odds ratios (OR) with corresponding 95% confidence intervals (CI). Factors with low frequency categories were included in the regression, and a sensitivity analysis excluding low-frequency categories was performed where there is evidence of inflated standard errors and ORs. This is a complete case analysis with an alpha level of 5% (*P* < 0.05) to be considered statistically significant.

## Results

There was a total of 964 responses to the survey, however, after excluding participants who did not fit the selection criteria and duplicate responses, there was a total of 928 eligible survey responses.

Participant characteristics are shown in Table 1. Responses were received from all states and territories of Australia, with the majority residing in major city areas. The median age of participants was 28 years [interquartile range 23–39]. Sixty three percent of trans adults reported a lifetime history of intentional self-harm (*n* = 577), while 43% reporting ever having attempted suicide (*n* = 394). This compares to a lifetime prevalence of self-injury in the Australian general population of 8.1% and previous suicide attempts of 3.3% [41, 42]. From univariate analysis, there was no statistically significant difference in the proportion of suicide (*p* = 0.6) or self-harm (*p* = 0.08) between different states of residence.

**Table 1:** Participant Characteristics

Parameter	Number of responses received	Frequency n(%)
*State of residence*	911	
Victoria		282 (31%)
New South Wales		195 (21%)
Queensland		143 (16%)
Western Australia		126 (14%)
South Australia		92 (10%)
Tasmania		37 (4%)
Australian Capital Territory		34 (4%)
Northern Territory		2 (< 1%)
*Location of residence (rural status)*	905	
Major city areas (Remoteness Area 1)		752 (83%)
Inner regional areas (Remoteness Area 2)		122 (14%)
Outer regional areas (Remoteness Area 3)		25 (3%)
Remote and Very Remote areas (Remoteness Area 4 and Remoteness Area5)		6 (< 1%)
*Age group (years)*	928	
18–24		289 (31%)
25–29		216 (23%)
30–39		193 (21%)
40–49		125 (13%)
50–59		71 (8%)
60–69		30 (3%
70–79		4 (< 1%)
*Presumed sex at birth*	928	
Female		520 (56%)
Male		403 (43%)
Intersex		5 (1%)
*Gender identity*	928	
Trans Man/Trans Male/Transmasculine		239 (26%)
Trans Woman/Trans Female/Transfeminine		202 (22%)
Female		140 (15%)
Gender Non-Binary		133 (14%)
Male		91 (10%)
Gender Queer		41 (4%)
Agender		20 (2%)
Gender Fluid		19 (2%)
Gender Neutral		11 (1%)
Other		30 (3%)
*Employment status*	928	
Employed on a full-time basis		274 (30%)
Employed on part-time or casual basis		224 (24%)
Home duties full time		13 (1%)
Student		176 (19%)
Retired		20 (2%)
Unemployed		177 (19%)
Other (freetext)		44 (5%)
*Depression and Anxiety*	914	
Depression		663 (85%)
Anxiety		613 (79%)
Discrimination*	927	
Discrimination from employment		304 (35%)
Discrimination from accessing healthcare		244 (28%)
Discrimination from government services		149 (17%)
Discrimination from housing		95 (11%)
Verbal Assault		584 (68%)
Physical Assault		200 (23%)
Domestic violence		133 (15%)
Difficulty accessing hormonal treatment*	905	
None		372 (44%)
Pathway to accessing hormones was too difficult		284 (33%)
Unable to find a doctor to prescribe		148 (17%)
Financial costs of prescriptions		124 (15%)
Financial costs of doctors appointments		156 (18%)
Other (specify)		100 (12%)
Access to Gender-Affirming Surgery*		
*People presumed male at birth*	384	
- Had feminising genital surgery		71 (18%)
- Desired feminising genital surgery in the future		243 (64%)
- No desire for feminising genital surgery		70 (18%)
*People presumed female at birth*	511	
- Had mastectomy or chest reconstructive surgery		159 (31%)
- Desired mastectomy or chest surgery		297 (58%)
- No desire for mastectomy or chest surgery		55 (11%)
*Member of Trans Peer Support Groups*	860	
Yes		689 (80%)
No		153 (18%)
Unsure/Prefer not to say		18 (2%)

*multiple responses allowed for this question so total responses do not sum to 100%

Variables which were associated with increased odds of a lifetime history of suicide attempts are shown in Table 2. Self-reported unemployment, desiring gender-affirming surgery in the future, depression, physical assault, and institutional discrimination were all associated with higher odds of reporting a previous suicide attempt. There was no association with location of residence (rural versus metropolitan), nor was access to trans support groups a protective factor. Being presumed male at birth was associated with lower odds of reporting a lifetime history of suicide attempts. Due to the low number of intersex individuals (*n* = 5), a valid odds ratio cannot be estimated and hence was not reported in Table 2. A sensitivity analysis was performed excluding those 5 participants and the results remains unchanged.

**Table 2:** Variables and association with a lifetime history of suicide attempts

Variable	Unadjusted OR (95% CI)	***P***	Adjusted OR (95% CI)	***P***
Location (Living outside of a major city area in Remoteness Areas 2–5).	0.97 (0.68, 1.38)	0.8	0.93 (0.61, 1.41)	0.7
Presumed Male at Birth	0.65 (0.50, 0.85)	0.002	0.62 (0.45, 0.85)	0.003
Unemployment	1.88 (1.35, 2.63)	0.0002	1.54 (1.04, 2.28)	0.03
Access to gender-affirming hormone therapy (difficulty accessing)	1.65 (1.25, 2.18)	0.0004	0.97 (0.70, 1.34)	0.8
Access to gender-affirming surgery (wanting in future)	1.71 (1.20, 2.43)	0.003	1.71 (1.13, 2.59)	0.01
Depression	4.64 (3.27, 6.58)	< 0.0001	3.43 (2.16, 5.46)	< 0.0001
Anxiety	2.85 (2.11, 3.84)	< 0.0001	1.13 (0.74, 1.73)	0.6
Access to Trans Support Group	0.92 (0.66, 1.30)	0.7	0.79 (0.54, 1.16)	0.2
Physical Assault	2.55 (1.85, 3.51)	< 0.0001	2.00 (1.37, 2.93)	0.0004
Institutional Discrimination	1.91 (1.47, 2.49)	< 0.0001	1.59 (1.14, 2.22)	0.007

*OR* odds ratio, Unadjusted OR (95% CI) from univariate Logistic regression; Adjusted OR (95% CI) from Logistic regression with all variables included (complete case analysis *n* = 785), mutually adjusted for each other

## Discussion

This large community survey provides preliminary insight into the factors associated with suicidality in the Australian trans community. Being unemployed, reporting a diagnosis of depression, desiring gender affirming surgery, a history of physical assault and experiences of institutional discrimination were all factors associated with increased odds of a lifetime history of suicide attempts. Being presumed male at birth was associated with lower odds of suicide attempt.

While the self-reported suicide attempt rate of trans participants is 10-times higher than that reported for the general Australian population, this rate converges with data on Australian trans youth and similar cohort studies conducted in Euro-Western settings [6, 41–43]. This pattern of convergence suggests that health disparities and systemic social inequities are not confined to a specific developmental time frame nor geographic locality. Notably, we found intentional self-harm rates (63%) were even higher than the rate of suicide attempt, but previous evidence has shown that in the Australian population, self-harm can occur in the absence of suicidal thoughts, often used as a means of managing difficult emotions [42]. While beyond the scope of the current analysis, it may be that persistent social exclusion and acts of erasure result in elevated feelings of shame, hopelessness and isolation-factors associated with self-harm [24–29].

Due to widespread cissexism and transphobia, physical assault is an all too common experience within the trans community. It was reported by 23% of respondents and was associated with a 200% increase in the odds of a lifetime suicide attempt. Physical assault has consistently been associated with poor mental health outcomes and a higher risk of suicide [19, 20, 44]. Critically, being physically assaulted because of a perpetrator’s transphobic prejudice is associated with a higher probability of suicide attempt than a physical assault not attributed to prejudice, or experiencing institutional discrimination alone without assault [45].

Additionally, experiences of institutionalised discrimination were reported at a high frequency. In our study, this included discrimination while accessing healthcare (including gender affirming healthcare), in employment, housing, and accessing government services. In a US-based study of 6450 trans people, an extraordinary 90% reported experiencing harassment, mistreatment or discrimination in workplaces, housing and in healthcare settings due to prejudice related to their trans-status or took actions such as hiding their identity to mitigate risk [3]. Specifically, service denial in healthcare has a profound impact correlated with elevated rates of attempted suicide [21]. Social and institutional discrimination has been found to negatively impact trans people’s mental health and has been consistently demonstrated to be a risk factor for attempted suicide, underscoring the need for multi-level interventions to enable timely, rights-based and culturally safe access to gender affirming and general healthcare, end discrimination and protect the trans population across every domain of life [18, 29, 46, 47].

In addition to discrimination, unemployment was associated with a 55% higher odds of lifetime suicide attempt. The trans unemployment rate of 19% is three times higher than the general Australian population (5.5%) [48]. In general population studies, unemployment and financial precarity has been linked to suicidality, with the length of unemployment compounding the risk of suicide [10–12]. The impact of employment on mental and physical health, socioeconomic status and quality of life is profound [49, 50]. Perceived stress in everyday life is known to increase the risk of unemployment, yet unemployment and sustained economic hardship can also directly negatively affect physical, psychological and cognitive functioning [51–54]. Poverty arising from unemployment may additionally limit an individual’s ability to access gender-affirming healthcare, particularly gender-affirming surgery which is associated with large out-of-pocket costs [3, 55]. Notably, there are many potential barriers to employment for trans people such as persistent challenges being affirmed and respected by employers and colleagues using the correct name, gender and pronouns, to being terminated, looked over for promotions and facing discrimination and violence at work, to discrimination in basic housing and healthcare and the impact of mental health conditions such as depression and anxiety on an individual’s ability to seek or maintain employment [29, 56]. Moreover, 35% reported perceived discrimination from employment, and whilst it was not directly assessed in the survey questions, workplace environments that expose individuals to discrimination have been found elsewhere to impact on an individual’s mental health and ability to maintain employment [29].

Self-reported lifetime diagnoses of depression were high in our participants, and this was associated with an over 300% increased odds of reporting a lifetime suicide attempt. Similarly, a lifetime history of major depressive disorder has been significantly associated with increased risk of suicidal ideation and attempted suicide in trans people worldwide [8, 9]. Depression in trans people is multifaceted, and there are various contributing factors; including discrimination, disclosure, social support, access to gender affirming healthcare, substance use and socioeconomic factors [57]. As such, strategies to lower the high rates of depression will need to be multifaceted, supported by accessible, specific and safe mental health support services for trans individuals, and improved access to gender affirming healthcare [58].

One of the biggest barriers reported by trans individuals is a lack of access to healthcare due to the lack of healthcare professionals skilled in gender affirming healthcare [59]. Access to gender-affirming surgery, in particular, poses significant barriers due to a lack of experienced surgeons, high cost, the lack of public funding and “gate-keeping” requirements, which can typically involve multiple, detailed assessments with two mental health professionals prior to surgery, even though, access to gender-affirming surgery has been shown to improve mental health and quality of life indicators for those who have undertaken a surgical intervention to affirm their gender [5, 33, 60]. We demonstrate that not being able to access surgery but desiring it, is associated with 73% increased odds of reporting a lifetime suicide attempt.

In an Australian study regarding surgery experiences and satisfaction, depression was reported in 34% of those individuals who had undergone at least some form of gender-affirming surgery, compared to 51.3% in those who desire but had not undergone surgery [33]. Our findings concur with previous research that those who want surgery but have yet to access it, are at significantly increased risk of suicide, while having access to desired surgery is a protective factor against suicidality. Greater training, programs and clinical supervision for surgeons already conducting or wishing to conduct gender affirming surgery, along with full public funding for all gender-affirming surgeries is critical to address this healthcare gap in access to such medically necessary interventions.

Interestingly our findings show that trans women and non-binary participants presumed male at birth appeared to have a lower odds of suicide attempt and the converse is true for trans men and non-binary participants presumed female at birth. Whilst suicide deaths in the Australian population occur at higher rates in those recorded as male, there is a higher rate of suicidal ideation and suicide attempt in those presumed female at birth [61]. Certainly studies assessing suicide attempts in the trans community have shown variable gender distributions and inferences are unclear [62].

Previous research suggests that a lack of social support is associated with higher odds of psychological distress and lifetime suicide attempts, and that social support from the trans community is a protective factor against suicidal ideation and suicide attempts [17, 63]. Contrary to those studies, our study indicates that there is no significant association between being part of a trans support group and suicide attempts. Notably, our survey did not ask about community connection which is different from being a member of a support group, nor did the survey assess other forms of social support, such as that from family and friends, which has been shown to be a protective factor [13, 14, 16, 64].

## Limitations

There are multiple limitations to this online study utilising a non-probability snowball sampling approach. The online-based recruitment may explain the proportion of younger participants and the views of older trans people may not be accurately reflected. There may be self-selection bias and not all areas of Australia were represented equally as recruitment was not targeted. There was a predominance of respondents in South-Eastern states, which may be related to physical promotion of the study at one event in Victoria and New South Wales. However, distribution of respondents was similar to a previous 2013 Western Australian-based survey [5]. Depression, self-harm and suicide attempts were self-reported. Hence, it is not possible to confirm diagnosis or determine how individuals define their experiences (e.g. what constitutes self-harm versus a suicide attempt; diagnosis of clinical depression). We did not study completed suicide, however suicide attempts are a risk factor for suicide and reflect significant distress experienced. The survey was also designed to broadly explore healthcare and wellbeing in the trans community and as such, did not focus extensively on mental health and suicidality. This survey was, however, a platform for trans people in Australia to express their experiences and opinions anonymously and honestly. It provides valuable insight on the health needs and wellbeing of a marginalised community.

## Conclusion

This large community survey highlights the high rates of attempted suicide, self-harm and depression in the trans community. Suicide attempts occur due to a complex interaction between socio-political, environmental, interpersonal and structural risk factors. Rather than suicidality perceived as inherent to the trans experience, trans people appear to exhibit higher rates of suicidality as a manifestation of healthcare inequities; discrimination, assault and barriers to accessing necessary gender affirming healthcare, including surgical intervention. Addressing these factors that contribute to suicidality and the mental health burden in the trans community must be made a priority. Dismantling barriers to gender-affirming healthcare, including wider availability of affordable surgery is paramount; as is tackling pervasive cissexism in order to reduce incidents of discrimination, stigmatization and violence. There is also an ongoing need to shift the discourse of the health and health needs of trans people away from a focus on risk and deficit, to align with a strength-based approach to illuminate factors that protect against suicidality and to promote resilience.

## Data Availability

The datasets used and/or analysed during the current study are available from the corresponding author on reasonable request.
