# Correction to: Factors associated with suicide attempts among Australian transgender adults

**DOI:** 10.1186/s12888-021-03491-w

**Published:** 2021-11-09

**Authors:** Sav Zwickl, Alex Fang Qi Wong, Eden Dowers, Shalem Yiner-Lee Leemaqz, Ingrid Bretherton, Teddy Cook, Jeffrey D. Zajac, Paul S. F. Yip, Ada S. Cheung

**Affiliations:** 1 Trans Health Research Group, Department of Medicine (Austin Health), The University of Melbourne, Melbourne, Victoria 3084 Australia; 2 College of Medicine and Public Health, Flinders University, Adelaide, South Australia 5042 Australia; 3 Department of Endocrinology, Austin Health, 145 Studley Road, Heidelberg, Victoria 3084 Australia; 4 ACON Health, Surry Hills, New South Wales Australia; 5 Centre for Suicide Research and Prevention, The University of Hong Kong, Hong Kong, Hong Kong, China

**Correction to: BMC Psychiatry 21, 81 (2021)**

**https://doi.org/10.1186/s12888-021-03084-7**

Following the publication of the original article [1], the authors provided clarifications, additional information, and changes in the text and tables. The changes have been highlighted with **bold typeface** and are shown in Additional file 1.

The original article [1] has been corrected.

## Supplementary Information

**Additional file 1.**
