# Supplementary material for: Correction to: Factors associated with suicide attempts among Australian transgender adults
Source: BMC Psychiatry. 2021 Nov 9;21:551. doi: 10.1186/s12888-021-03491-w (PMC8579568; doi:10.1186/s12888-021-03491-w)
Supplement: Supplementary file 1 — Additional file 1. [file 12888_2021_3491_MOESM1_ESM.docx]

Abstract

Results: Of 928 participants, **73%** self-reported a lifetime diagnosis of depression, 63% reported previous self-harm, and 43% had attempted suicide. Higher odds of reporting a lifetime history of suicide attempts were found in people who were; unemployed (adjusted odds ratio (aOR) **1.54** (**1.04, 2.28**), p=0.03), had a diagnosis of depression (aOR **3.43** (**2.16, 5.46**), p<0.001), desired gender affirming surgery in the future (aOR **1.71** (**1.13, 2.59**), p=0.01), had experienced physical assault (aOR **2.00** (1.37, **2.93**), p<0.001) or experienced institutional discrimination related to their trans status (aOR 1.59 (1.14, **2.22**), p=0.007).

Conclusion: Suicidality is associated with **desiring gender affirming surgery in the future**, gender based victimisation and institutionalised cissexism.

Methods

We specifically assessed if the following **10** factors were risk or protective factors for a positive(‘yes’) response for a lifetime history of attempted suicide.

5) **Desire for** gender affirming surgery **in the future**. Participants indicated whether they wanted gender affirming surgery someday, had already had surgery or did not want surgery, **from the four options – bilateral mastectomy/chest reconstruction surgery, breast augmentation, bottom surgery, voice surgery**. **Those** that desired **at least one type of** gender affirming surgery **were** compared with other groups that did not.

**7) Self-reported diagnosis of anxiety. Participants were asked if they had ever been medically diagnosed with anxiety (yes/no).**

**8)** Access to trans support groups. Participants were asked if they were a member of any trans support groups, including on social media (yes/no or unsure).

**9)** Participants were asked ‘Because of your trans status have you ever experienced any of the following (select all that apply)?’ with multiple choice options of ‘Discrimination from employment (i.e. lost a job or overlooked for a job)’, ‘Discrimination from housing (i.e. denied a rental application)’, ‘Discrimination from accessing healthcare’, **and** ‘Discrimination from government services (i.e. Centrelink)’, ‘Physical assault’, ‘Verbal abuse’, ‘Domestic violence’, and ‘None’.

**10)** Physical assault. Participants indicated whether they had ever experienced physical assault because of their trans status (yes/no).

Logistic regression was used to estimate the effects of the **10** factors listed above on the risk of attempted suicide. The **10** factors considered in the regression were selected prior to performing the analysis on the basis of previous known risk factors for suicidal behaviour.

Results

**Access to and desire for gender affirming surgeries are presented in Table 2.**

Variables which were associated with increased odds of a lifetime history of suicide attempts are shown in **Table 3**. There was no association with **anxiety, difficulty accessing hormones or** location of residence (rural versus metropolitan), nor was access to trans support groups a protective factor. Due to the low number of intersex individuals (n=5), a valid odds ratio cannot be estimated and hence was not reported in **Table 3**.

Discussion

Due to widespread cissexism and transphobia, physical assault is an **all-too-common** experience within the trans community. It was reported by **21%** of respondents and was associated with a **100%** increase in the odds of a lifetime suicide attempt.

In addition to discrimination, unemployment was associated with a **54%** higher odds of lifetime suicide attempt.

Moreover, **33%** reported perceived discrimination from employment, and whilst it was not directly assessed in the survey questions, workplace environments that expose individuals to discrimination have been found elsewhere to impact on an individual’s mental health and ability to maintain employment[[29](#_ENREF_29)].

Self-reported lifetime diagnoses of depression were high in our participants, and this was associated with an over **200%** increased odds of reporting a lifetime suicide attempt.

**Anxiety, which is highly prevalent in the trans community, was not significantly associated with lifetime suicide attempt after adjustment, suggesting that the association is influenced by other confounders, such as depression.  This is inline with some general population studies that have found that anxiety disorder alone is not associated with suicidality [**[**59**](#_ENREF_59)**].**

**We demonstrate that trangender individuals who desire gender affirming surgery in the future experience 71% increased odds of reporting a lifetime suicide attempt. This is likely related to a number of intrapersonal and interpersonal factors, and barriers to healthcare access. Those individuals who desire gender affirming surgery generally experience body and/or social dysphoria related to that part of their body, resulting in mental health distress. Gender affirming surgeries may result in significant body changes that increase the likelihood that trans individuals will be read and understood by others as their affirmed gender. Those who desire but are yet to access surgeries may experience higher rates of misgendering, discrimination and violence due to gender non-conformity or ambiguous appearance [**[**3**](#_ENREF_3)**,** [**60**](#_ENREF_60)**], which in turn may have an impact on mental health.**

**Access to gender-affirming surgery has been shown to improve mental health and quality of life indicators for those who have undertaken a surgical intervention to affirm their gender. [**[**5**](#_ENREF_5)**,** [**33**](#_ENREF_33)**,** [**61**](#_ENREF_61)**] In an Australian study regarding surgery experiences and satisfaction, depression was reported in 34% of those individuals who had undergone at least some form of gender-affirming surgery, compared to 51% in those who desired but had not undergone surgery. [**[**33**](#_ENREF_33)**] Our findings concur with previous research that those who want surgery but have yet to access it, are at significantly increased risk of suicide.**

**Desire for gender affirming surgery in the future may also be related to healthcare access.** [[62](#_ENREF_62)] Access to **gender-affirming** surgery, in particular, poses significant barriers due to a lack of experienced surgeons, high cost, the lack of public funding and “gate-keeping” requirements, which can typically involve multiple, detailed assessments with two mental health professionals prior to surgery. **Barriers to access, may therefore also contribute to mental health distress and suicality, as individuals are faced with long, complicated and often prohibitively expensive options for gender affirming surgeries.**

**In the Australian general population, the rates of suicide tend to increase with increasing rurality. This is commonly associated with several factors, including fewer essential services such as healthcare and mental health support. [**[**65**](#_ENREF_65)**,** [**66**](#_ENREF_66)**] This study however, showed no statistically significant difference in lifetime suicide attempt between trans people living in inner city areas and those living in regional and remote areas. Protective factors that might mitigate the expected association between rurality and suicidality include reasons for living, the individual’s resilience and ability to self-regulate suicidal thoughts and feelings, familial and social support and optimism. [**[**67**](#_ENREF_67)**,** [**68**](#_ENREF_68)**] However, there is relatively little research directly examining protective factors in the trans population and the experience of trans individuals and communities in regional and remote areas, an effect termed the ‘metronormative’ bias of trans research. [**[**69**](#_ENREF_69)**] Seminal qualitative research conducted in the USA illuminates how trans experiences of resilience in regional and rural places rests upon other social positions (e.g., race, queerness, disability and sexuality). [**[**70**](#_ENREF_70)**]**

**Not all trans people desire gender affirming hormones in their transition. However, for those people who do, the ability to access hormones reduces mental distress. [**[**31**](#_ENREF_31)**,** [**32**](#_ENREF_32)**] The highest rates of depression in trans people are in those who want hormones but have yet to use them or are unable to access them. [5] Despite the strong link between depression and suicidality, this study found no significant difference in suicidality solely based on access to hormones. Given that there may be many confounding factors that impact mental health independently of hormone therapy, such as access to other gender affirming medical procedures and psychotherapy, as well as social support, it is difficult to determine the independent effects of hormone therapy on quality of life. [**[**32**](#_ENREF_32)**] There is also evidence that any form of gender affirming transition is beneficial, such as social transition and social acceptance. [**[**67**](#_ENREF_67)**]**

Conclusion

Suicide attempts occur due to a complex interaction between socio-political, environmental, interpersonal and structural risk factors. Rather than suicidality perceived as inherent to the trans experience, trans people appear to exhiit higher rates of suicidality as a manifestation of **social discrimination.**

**Table 1. Participant Characteristics**

| **Parameter** | **Number of responses received** | **Frequency n(%)** |
| --- | --- | --- |
| *State of residence*  Victoria  New South Wales  Queensland  Western Australia  South Australia  Tasmania  Australian Capital Territory  Northern Territory | 911 | 282 (31%)  195 (21%)  143 (16%)  126 (14%)  92 (10%)  37 (4%)  34 (4%)  2 (<1%) |
| *Location of residence (rural status)*  Major city areas (Remoteness Area 1)  Inner regional areas (Remoteness Area 2)  Outer regional areas (Remoteness Area 3)  Remote and Very Remote areas (Remoteness Area 4 and Remoteness Area5) | 905 | 752 (83%)  122 (**13**%)  25 (3%)  6 (<1%) |
| *Age group (years)*  18-24  25-29  30-39  40-49  50-59  60-69  70-79 | 928 | 289 (31%)  216 (23%)  193 (21%)  125 (13%)  71 (8%)  30 (3%  4 (<1%) |
| *Presumed sex at birth*  Female  Male  Intersex | 928 | 520 (56%)  403 (43%)  5 (1%) |
| *Gender identity*  Trans Man/Trans Male/Transmasculine  Trans Woman/Trans Female/Transfeminine  Female  Gender Non-Binary  Male  Gender Queer  Agender  Gender Fluid  Gender Neutral  Intersex  Other | 928 | 239 (26%)  202 (22%)  140 (15%)  133 (14%)  91 (10%)  41 (4%)  20 (2%)  19 (2%)  11 (1%)  2 (**<1%**)  30 (3%) |
| *Employment status*  Employed on a full-time basis  Employed on part-time or casual basis  Home duties full time  Student  Retired  Unemployed  Other (freetext) | 928 | 274 (30%)  224 (24%)  13 (1%)  176 (19%)  20 (2%)  177 (19%)  44 (5%) |
| *Depression and Anxiety*  Depression  Anxiety | 914 | 663 (**73**%)  613 (**67**%) |
| *Discrimination**  Discrimination from employment  Discrimination from accessing healthcare  Discrimination from government services  Discrimination from housing  Verbal Assault  Physical Assault  Domestic violence | 927 | 304 (**33**%)  244 (**26**%)  149 (**16**%)  95 (**10**%)  584 (**63**%)  200 (**21**%)  133 (**14**%) |
| *Difficulty accessing hormonal treatment**  None  Pathway to accessing hormones was too difficult  Unable to find a doctor to prescribe  Financial costs of prescriptions  Financial costs of doctors appointments  Other (specify) | 905 | 372 (**41**%)  284 (**31**%)  148 (**16**%)  124 (**14**%)  156 (**17**%)  100 (**11**%) |
|  |  |  |
| *Member of Trans Peer Support Groups*  Yes  No  Unsure/Prefer not to say | 860 | 689 (80%)  153 (18%)  18 (2%) |

*multiple responses allowed for this question so total responses do not sum to 100%.

**Table 2. Access to and Desire for Gender Affirming Surgery**

|  | **Number of responses recieved** | **Have had, n (%)** | **Want someday, n (%)** | **Don’t want, n (%)** |
| --- | --- | --- | --- | --- |
| **Surgical procedures in people presumed male at birth** | | | | |
| **Breast augmentation** | **362** | **32 (9)** | **196 (54)** | **134 (37)** |
| **Genital reconscrutive surgery** | **384** | **71 (18)** | **243 (63)** | **70 (18)** |
| **Facial feminization surgery** | **372** | **23 (6)** | **235 (63)** | **114 (31)** |
| **Voice surgery** | **348** | **6 (2)** | **149 (43)** | **193 (55)** |
| **Surgical procedures in people presumed female at birth** | | | | |
| **Chest reconstructive surgery / mastectomy** | **511** | **159 (31)** | **297 (58)** | **55 (11)** |
| **Genital reconscrutive surgery** | **481** | **10 (2)** | **213 (44)** | **258 (54)** |
| **Voice surgery** | **405** | **1 (<1)** | **15 (4)** | **389 (96)** |

***multiple responses allowed for this question so total responses do not sum to 100%.**

**Table 3. Variables and association with a lifetime history of suicide attempts**

| **Variable** | **Unadjusted**  **OR (95% CI)** | ***P*** | **Adjusted**  **OR (95% CI)** | ***P*** |
| --- | --- | --- | --- | --- |
| Location (Living outside of a major city area in Remoteness Areas 2 – 5). | 0.97 (0.68, 1.38) | 0.8 | 0.93 (0.61, 1.41) | 0.7 |
| Presumed Male at Birth | 0.65 (0.50, 0.85) | 0.002 | 0.62 (0.45, 0.85) | 0.003 |
| Unemployment | 1.88 (1.35, 2.63) | 0.0002 | 1.54 (1.04, 2.28) | 0.03 |
| Access to gender-affirming hormone therapy (difficulty accessing) | 1.65 (1.25, 2.18) | 0.0004 | 0.97 (0.70, 1.34) | 0.8 |
| Access to gender-affirming surgery (wanting in future) | 1.71 (1.20, 2.43) | 0.003 | 1.71 (1.13, 2.59) | 0.01 |
| Depression | 4.64 (3.27, 6.58) | <0.0001 | 3.43 (2.16, 5.46) | <0.0001 |
| Anxiety | 2.85 (2.11, 3.84) | <0.0001 | 1.13 (0.74, 1.73) | 0.6 |
| Access to Trans Support Group | 0.92 (0.66, 1.30) | 0.7 | 0.79 (0.54, 1.16) | 0.2 |
| Physical Assault | 2.55 (1.85, 3.51) | <0.0001 | 2.00 (1.37, 2.93) | 0.0004 |
| Institutional Discrimination | 1.91 (1.47, 2.49) | <0.0001 | 1.59 (1.14, 2.22) | 0.007 |

OR = odds ratio; Unadjusted OR (95% CI) from univariate Logistic regression; Adjusted OR (95% CI) from Logistic regression with all variables included (complete case analysis n=785), mutually adjusted for each other.
